# Developing a cognitive model of advanced teacher professional vision for understanding processes of noticing and reasoning

**DOI:** 10.3389/fpsyg.2026.1769094

**Published:** 2026-06-17

**Authors:** Tina Seidel, Ricardo Böheim, Christian Kosel

**Affiliations:** Department Educational Sciences, TUM School of Social Sciences and Technology, Technical University of Munich, Munich, Germany

**Keywords:** eye tracking, noticing, reasoning, student engagement, teacher expertise, teacher professional vision, visual attention

## Abstract

Teacher professional vision (PV) describes teachers’ situation-specific ability to notice and reason about pedagogically relevant classroom events – encompassing both instructional learning processes and classroom management interactions. PV comprises two core processes: noticing (the selective perception of relevant classroom cues) and reasoning/interpretation (the knowledge-based process of making sense of what has been noticed). Previous research has consistently shown that novice and expert teachers differ systematically in their PV skills and that these skills can be fostered through targeted professional development. While existing models of professional vision have provided important conceptual distinctions between noticing and reasoning, a theory-based specification of the underlying cognitive processes remains limited. In this conceptual contribution, we address this gap by introducing a cognitive processing model of teacher professional vision (PV-CP model) that aligns PV research with central assumptions from educational cognitive psychology and expertise research. The model differentiates four core processes: two noticing-related processes (information selection and breadth of the visual field) and two reasoning-related processes (cognitive organizing and integrating). Drawing on recent empirical studies of PV, predominantly using eye-tracking methods, we synthesize systematic differences between novice and experienced teachers across these processes. Experienced teachers are characterized by rapid, knowledge-guided information selection, holistic classroom monitoring, and the ability to organize and integrate visual information into coherent mental models, enabling accurate interpretation and adaptive decision-making. Building on the PV-CP model, we further discuss implications for the design of professional vision training in teacher education. In particular, we discuss video-based learning environments, eye movement modeling examples, and verbal expert self-explanations and outline how these approaches may support targeted development of noticing and reasoning processes. By providing a cognitively grounded process model, this contribution advances theoretical integration in PV research and offers a systematic framework for future empirical studies and instructional interventions.

## 1 Understanding teacher professional vision: a cognitive perspective

Imagine a teacher leading a classroom discussion. While one student at the front is presenting their solution on the board, several other events unfold simultaneously: a pair of students in the back are quietly comparing their notes, another student hesitates before answering a peer’s question, and two others exchange glances that suggest confusion. In this specific moment, a teacher simultaneously processes multiple layers of activity to decide whether to probe the hesitant student, clarify the task for the confused pair, or redirect attention to maintain focus on the presenting student. In other words, a teacher notices several learning-relevant events and interprets them in ways that inform adequate pedagogical action. Teachers require what educational researchers refer to as professional vision (PV): the ability to notice and reason about classroom events in situation-specific ways. PV has become a central construct in educational research, highlighting systematic skill differences between novices and experts and underscoring the importance of professional knowledge and deliberate practice for demonstrating advanced professional vision skills (Gegenfurtner et al., 2011; Gegenfurtner and Stahnke, 2024; Kosel et al., 2024). While prior PV models have provided rich descriptions about components of PV and their relationship with professional dispositions and performance (e.g., Seidel and Stürmer, 2014; Seidel et al., 2024; Blömeke et al., 2015)–a critical gap remains: the lack of a theory-based conceptual model that specifies and links processes of PV to the underlying human cognitive system.

In this conceptual paper, we address this gap by adopting a cognitive educational psychology perspective and aligning PV-related processes with their corresponding cognitive structures. Specifically, we introduce the Professional vision cognitive processing model (PV-CP model), a model that differentiates between four core cognitive processes–two related to noticing (information selection and breadth of visual field) and two related to reasoning (cognitive organizing and integrating). This model builds on Seidel and Stürmer (2014) PV framework as well as models of visual expertise (Gegenfurtner, 2024) and synthesizes recent empirical research, offering a conceptual alignment of teacher professional vision research with expertise research in educational cognitive psychology. The PV-CP model focuses on intra-individual cognitive processes and does not directly model external contextual factors. These contextual dimensions, while highly relevant to understanding professional vision in practice (Duvivier, 2025; Duvivier et al., 2026; Gegenfurtner, 2020), are treated as inputs to and outputs of the cognitive processing system rather than as components of the model itself. PV is fundamentally contextually situated, as it develops through teachers’ selective attention to and interpretation of events in specific classroom situations (Gegenfurtner, 2024). PV processes are assumed to depend on situational, social, and material features of classrooms, meaning that what teachers notice and interpret depends on the specific instructional context. Moreover, the model provides a robust foundation for future studies, as it supports the systematic selection and targeting of relevant PV processes. In doing so, this conceptual paper not only formalizes the cognitive underpinnings of PV but also proposes concrete directions for future research and PV training.

Professional vision is commonly understood as a characteristic of developed teacher expertise, describing the ability to perceive, interpret, and respond to classroom interactions in ways that are instructionally meaningful and pedagogically informed (Santagata et al., 2021; Seidel and Stürmer, 2014; Sherin and van Es, 2009). It is a learnable skill that develops through knowledge and professional dispositions to support adaptive teaching behavior in the classroom (Seidel et al., 2024). Throughout this paper, “advanced professional vision” refers specifically to the process profile described by the PV-CP model: the characteristic pattern of fast, knowledge-guided information selection, holistic classroom monitoring, and coherent organizing and integrating that distinguishes expert from novice teachers. This definition anchors ‘advanced’ to measurable process characteristics rather than to proxy measures such as years of experience. It consists of two main components: selective attention (noticing) and knowledge-based reasoning. Noticing is commonly defined as a perceptual-cognitive lens through which teachers selectively attend to relevant events of classroom interactions. It involves identifying pedagogically significant classroom events and separating them from events that are less relevant to the ongoing instruction. Reasoning is the interpretive process of making sense of what is noticed using professional knowledge, including pedagogical content knowledge, content knowledge and knowledge about effective teaching and student learning (Shulman, 1986). Reasoning is essential to integrate and organize processed information into meaningful ways for deeper understanding. Thereto, teachers are enabled to respond thoughtfully and adaptively to student behavior.

Influential PV models are grounded in Goodwin’s anthropological framing (1994), including Sherin’s distinction between selective attention and knowledge-based reasoning (Sherin and van Es, 2009), Blömeke’s perception–interpretation–decision-making model (Blömeke et al., 2015) and Seidel’s threefold reasoning structure of description, explanation, and prediction (Seidel and Stürmer, 2014). Recently, PV research has adopted eye-tracking technology, extending these PV models by shifting attention to the underlying visual and cognitive processes (Gegenfurtner et al., 2023; Lachner et al., 2016; Seidel et al., 2024). Among current developments, the Cognitive Theory of Visual Expertise (CTVE; Gegenfurtner et al., 2023; Gegenfurtner, 2024) is a direct theoretical basis of the present work. The CTVE accounts for expert visual performance across professional domains through eight cognitive processes operating across two memory stores – the visual register and long-term working memory – and grounds expertise in well-developed schemata held in long-term working memory. The PV-CP model shares parts this well-established cognitive psychology architecture and builds on it. In addition, it reorganizes it around the noticing–reasoning structure that has organized professional vision research since Goodwin (1994) and Seidel and Stürmer (2014) and around the situational demands of classroom teaching.

Research on teacher professional vision draws on multiple theoretical traditions and employs a range of terms – professional vision, teacher noticing, situation-specific skills, perception, interpretation – that are often used interchangeably but carry meaningfully different theoretical commitments across frameworks (König et al., 2022). To avoid confusions, we specify how these terms are used in the present paper. We use professional vision as the overarching construct, following Seidel and Stürmer (2014) and Sherin and van Es (2009), to refer to the situation-specific cognitive ability to perceive and interpret pedagogically relevant classroom events. Within this construct, we use noticing in a narrower and more specific sense than is common in parts of the literature: noticing refers here specifically to the perceptual-attentional processes through which teachers selectively encode visual classroom information – corresponding to the two PV-CP processes of information selection and breadth of the visual field. This is deliberately narrower than frameworks that use noticing to encompass both perception and interpretation (e.g., Sherin and van Es, 2009; Jacobs et al., 2010), and narrower than the full perception-interpretation-decision cycle described by Blömeke et al. (2015). We use reasoning to refer to the knowledge-based interpretive processes of cognitive organizing and integrating, which operate on the output of noticing. This two-level structure – noticing as perceptual input, reasoning as cognitive interpretation – follows Seidel and Stürmer (2014) and is the definitional architecture of the PV-CP model. We also clarify the use of the term expertise in this paper. Rather than treating expertise as a threshold defined by years of teaching experience – an operationalization that has been repeatedly criticized as theoretically underspecified (König et al., 2022; Cortina et al., 2024) – we define expertise in terms of a characteristic process profile: fast, knowledge-guided information selection; holistic classroom monitoring; and the capacity to organize and integrate observed events into coherent, adaptive mental models. Comparisons between novice and expert teachers throughout the paper should be understood as comparisons along this process-defined continuum, not as categorical distinctions based on experience alone.

**FIGURE 1:**
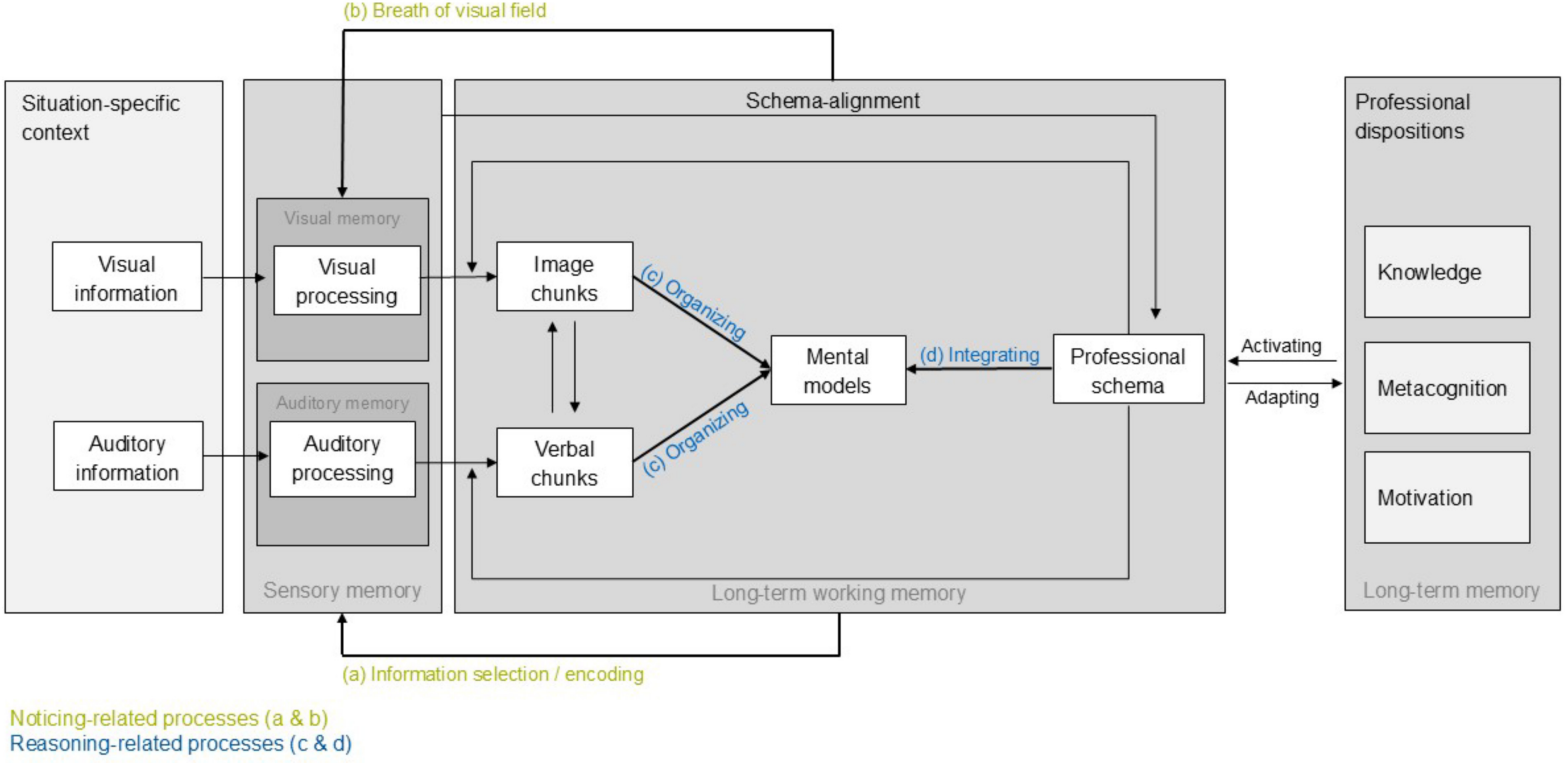
Professional vision cognitive processing model (PV-CP model).

In line with this development, we introduce conceptual considerations based on the original model of Seidel and Stürmer (2014) and a synthesis of recent empirical research in the field: a cognitive processing model of PV (PV-CP model) that differentiates between four core processes, unpacking the underlying visual and cognitive mechanisms. As depicted in Figure 1, noticing-related processes include information selection and breadth of the visual field, while reasoning-related processes include cognitive organizing and integrating. These processes develop over time, with novices initially attending more salient cues, while experts are efficiently integrating multiple streams of classroom information into coherent and organized pedagogical reasoning. Consequently, underlying visual and cognitive processes differ fundamentally between novice and expert teachers. The model follows a multi-memory store logic as established in cognitive psychology, where sensory memory captures incoming information, and long-term working memory supports reasoning and decision-making (Atkinson and Shiffrin, 1977; Ericsson and Kintsch, 1995; Mayer, 2014). The PV-CP model is grounded in the same multi-memory store logic stemming from cognitive psychology theories as the CTVE (Gegenfurtner et al., 2023; Gegenfurtner, 2024) and shares with it the differentiation of perceptual and interpretive processing that has organized cognitive accounts of visual expertise. The two frameworks operate, however, at different levels of analysis. The CTVE specifies the general cognitive processes through which visual expertise is realized across professional domains. The PV-CP specifies how these cognitive resources are organized within the situated structure of classroom interaction – around pedagogical scripts, dialogic patterns, and the noticing–reasoning architecture established in professional vision research (Seidel and Stürmer, 2014). Its four processes are defined within this structure: information selection and breadth of the visual field at the noticing level, cognitive organizing and integrating at the reasoning level. Two structural claims further distinguish the model: noticing and reasoning are conceived as reciprocal rather than sequential, and expert professional vision is conceived as the capacity to shift adaptively between automated and deliberate processing across aligned and non-aligned teaching situations.

Furthermore, PV-CP model focuses on the intra-individual cognitive processes underlying professional vision and does not model the behavioral and interactional dimensions of noticing and reasoning. Several important contributions have highlighted that professional vision also involves active behaviors through which teachers shape what becomes noticeable – including probing questions, strategic redirection, and deliberate attention-focusing interventions (van Es and Sherin, 2021; Scheiner, 2021; Gegenfurtner, 2024). These behavioral processes are not modeled as components of the PV-CP framework. Rather, the model provides the cognitive-level account that such behavioral accounts pre suppose: it specifies the internal processes that drive when, why, and how effectively a teacher engages in active information-seeking or shaping behavior. The PV-CP model and interactive accounts of professional vision thus operate at complementary levels of analysis and should be understood as mutually informative rather than competing.

Importantly, within the PV-CP model, expertise is not operationalized as years of teaching experience, but rather as a characteristic pattern of advanced professional vision processes – including fast, knowledge-guided information selection, holistic classroom monitoring, and the capacity to organize and integrate observed events into coherent mental models (see Section “2 The development of advanced PV skills: recent insights into PV-processing differences between experienced and novice teachers”). This process-oriented conceptualization of expertise is a foundational assumption of the model, not merely an empirical criterion for group comparisons.

While prior research has predominantly focused on descriptive expertise differences in noticing and reasoning separately, few studies have investigated the interplay between noticing- and reasoning-related PV processes (exceptions are Muhonen et al., 2021; Seidel et al., 2021). The PV-CP model addresses this gap by making explicit assumptions about how these processes relate to one another. In terms of processing direction, noticing-related processes are conceived as temporally and functionally prior: information selection and breadth of the visual field determine which perceptual input becomes available for subsequent cognitive processing. Reasoning processes of organizing and integrating then operate on this perceived input to construct and refine mental models of the classroom situation. However, this sequential direction does not imply a linear, one-way relationship. Consistent with the multi-memory store logic underlying the model (Ericsson and Kintsch, 1995; Mayer, 2014), reasoning-related structures – particularly well-developed schemata and mental models stored in long-term working memory – exert top-down regulatory effects on noticing processes. Activated professional knowledge shapes what teachers attend to and how broadly they scan the classroom, meaning that reasoning-level knowledge structures feed back into information selection and visual field breadth. The overall relationship between noticing and reasoning is therefore best characterized as reciprocal: noticing provides the visual input for reasoning, while reasoning-level knowledge continuously shapes what is noticed. This bidirectional relation is a core structural assumption of the PV-CP model and distinguishes it from frameworks that treat noticing and reasoning as independent or merely sequential stages.

Professional dispositions are assumed to show direct effects on noticing-related processes by functioning as top-down regulatory inputs that guide selective attention, prior to and independently of reasoning. In addition, dispositions shape reasoning-related processes more deliberately, by activating respective cognitive schema that structure how noticed events are interpreted and connected to professional knowledge. The two pathways are not mutually exclusive: the same determinant may simultaneously regulate what is attended to and how it is subsequently reasoned about, though the mechanisms and time scales involved differ.

In the following, we summarize expertise differences as they are expected according to the model from a conceptual perspective, while a detailed summary of previous research findings is given in the section following the model description.

### 1.1 Noticing processes

a) Information selection: Information selection refers to the encoding of visual and auditory cues such as a student raising a hand, chatting to a peer, or looking confused. This process is guided by prior knowledge and experience, which determines which cues are noticed and which are filtered out (Gegenfurtner, 2024). In addition, motivational factors and meta-cognitive dispositions guide attentional processes, such as goals that teachers might have for their students (Böheim et al., 2026; Daumiller et al., 2025).

- Novices: Novices tend to be driven by bottom-up processing, without being strongly guided in selecting information by activated schema and dispositions (Grub et al., 2020; van den Bogert et al., 2014). Thereto, novices are attending to the ongoing stream of information and select information based on more salient cues (Seidel et al., 2021). These cues, however, are not always pedagogically relevant (e.g., a student dropping a pencil or laughing). They are likely to miss subtle signals such as a student’s hesitation to speak.
- Experienced: Experienced teachers’ noticing is characterized by knowledge-related attentional control (Cortina et al., 2015; Huang et al., 2023; Kosel et al., 2021), wherein stored professional knowledge and dispositions are activated to guide attention toward behavioral cues with instructional relevance. For instance, when scanning the classroom, an experienced teacher may quickly pick up on a student’s puzzled expression, recognizing it as a sign of misunderstanding.

b) Breadth of the visual field: Classroom monitoring depends on both foveal (central) and parafoveal (peripheral) vision. Teachers need to attend not only to the student currently speaking but also to parallel activities across the entire classroom (Kosel et al., 2021).

- Novices: Often fixate narrowly on the student they are directly interacting with, thereby overlooking peripheral behaviors such as students quietly disengaging at the back (Keskin et al., 2023; Mendes et al., 2025)
- Experienced professionals: Use holistic scanning strategies, monitoring both central and peripheral zones. For example, while listening to one student’s response, they may simultaneously notice two students in the corner exchanging answers and decide to intervene if it disrupts learning (Cortina et al., 2015; Sheridan and Reingold, 2017).

### 1.2 Reasoning processes

Once relevant information has been selected, teachers engage in cognitive reasoning processes that draw on long-term working memory (Ericsson and Kintsch, 1995).

c) Organizing: Organization involves the process of categorizing a stream of ongoing information as it occurs in a classroom into meaningful units or pedagogical categories (Stern and Shavelson, 1983). This process organizes noticed information into distinctive events and helps to build a coherent mental model (Baddeley and Hitch, 2018; Ericsson and Kintsch, 1995) that serves as a basis for further interpretation.

- Novices: Novices organize information at a superficial level by constructing mental units that rely strongly on the more superficial cues that they have attended to initially (“off-task student in front row”).
- Experienced professionals: Organize the noticed stream of information into meaningful units and mark them with case-based pedagogical categories (“the student has shown a behavior of looking somewhat confused, chatting with the neighbor, pointing at the task sheet,…, so this set of behavior might be a sign of instructional unclarity”). Organizing processes, thereto, are indicated by precise observations and descriptions of events (Seidel and Stürmer, 2014) and indicative categorizations into higher order units (such as instructional unclarity).

d) Integrating: Integration refers to the process of linking professional knowledge and experiences to make sense of the initially organized mental models. Thereto, it further facilitates mental model building by making organized information units meaningful and interpretable. Integrating processes facilitate explanations and predictions (Seidel and Stürmer, 2014) and, in doing so, provide the cognitive basis from which pedagogical decision-making can proceed (Stern and Shavelson, 1983) – though decision-making itself, as a behavioral response process, lies beyond the scope of the present model.

- Novices: Novices systematically lack well-developed schemas and, thereto, often fail to integrate processed information in meaningful ways. Their interpretations as indicators for integrative processes often remain on a generic level, with tendencies to overgeneralize (“classes are always inattentive on Monday mornings”), or to attribute causes to individual students (“this student does not like mathematics”).
- Experienced professionals: Experienced professionals integrate activated professional knowledge and dispositions into organized mental model units, enabling them to meaningfully interpret observed classroom events in form of explanatory and predictive reasoning. For example, noticing a quiet student who consistently avoids participation, an experienced professional may infer possible underlying causes (e.g., lack of confidence, language barriers) as a basis for deciding on further pedagogical actions. Taken together, cognitive organizing and integrating constitute the interpretive core of professional vision. These interpretive processes provide the cognitive basis from which pedagogical decision-making and instructional action can proceed; however, the PV-CP model deliberately focuses on the cognitive processes of perception and interpretation and does not explicitly model downstream decision-making or pedagogical action.

The PV-CP model acknowledges that professional vision processes are not solely determined by cognitive knowledge structures, but are also shaped by teachers’ dispositional characteristics, including motivation and metacognition. Most directly supported by recent empirical evidence are motivational factors (Böheim et al., 2026; Daumiller et al., 2025). Teachers’ student-oriented goals are associated with their visual attention and the depth of their reasoning, suggesting that activated goals play an important role in information selection by directing attention toward particular student behaviors and activating cognitive schema that allow teachers to organize and integrate observed events in a more elaborated, knowledge-based manner. However, this evidence base is still emerging and the observed associations should not yet be interpreted as established causal mechanisms. Initial evidence further suggests that metacognitive dispositions such as self-monitoring strategies are more frequently used by experienced teachers when processing classroom situations (Gegenfurtner, 2020; Metallidou, 2009). On a theoretical basis, Blömeke et al. (2015, 2022) highlight the role of epistemic beliefs as relevant inputs to PV processes, even though the mechanisms through which beliefs shape these processes remain to be empirically investigated. Although the PV-CP model considers these determinants as highly relevant, the empirical basis for specifying their mechanisms remains limited, which also reflects the broader state of a field in which the cognitive and behavioral processes linking motivational dispositions to teaching behaviors are still poorly understood (Lazarides and Dresel, 2025).

## 2 The development of advanced PV skills: recent insights into PV-processing differences between experienced and novice teachers

The proposed PV-CP model builds on recent advancements in teacher expertise research describing systematic differences between experienced and novice teachers in knowledge-based noticing and reasoning. A comprehensive meta-analytic synthesis of eye-tracking evidence on teacher professional vision has recently been provided by Keskin et al. (2024), organized around the process categories of the Cognitive Theory of Visual Expertise. The present section serves a distinct and complementary purpose: it organizes existing empirical findings – including eye-tracking, verbal protocol, and behavioral judgment studies – specifically around the four processes of the PV-CP model, thereby demonstrating that the model’s process architecture is grounded in and supported by the existing evidence base.

### 2.1 Advanced noticing-related processes: knowledge-guided attentional control and holistic classroom monitoring

Studies investigating noticing-related PV processes suggest that experienced teachers tend to show more focused attention, concentrating on areas with relevant information, while novice teachers display more dispersed attention across the classroom (Wolff et al., 2016; Kosel et al., 2021; Kim and Yang, 2016; Stahnke and Blömeke, 2021). For example, Huang et al. (2023) analyzed data from mobile eye-tracking across 50 teachers and found that experienced teachers showed stronger selective attention, with fewer fixations on task-irrelevant objects and more attention directed toward students and instructional materials. In addition, experts distributed their gaze across a wider area of the classroom, suggesting a broader monitoring capacity compared to novices. Based on the PV-CP model, these processes can be explained by experts’ sophisticated declarative and procedural knowledge and the activation of routinized schema that allow efficient and dynamic attention shifts while keeping an overview of the entire classroom. These results exemplify how professional knowledge and experience foster advanced noticing-related PV processes of fast information selection and extended breadth of visual field. Similar results have been found in other studies, highlighting that novices often focus on non-instructional areas, whereas experts prioritize students in their gaze (McIntyre et al., 2019; Kim et al., 2012) and show a more even distribution of visual attention across students (Cortina et al., 2015).

### 2.2 Advanced reasoning-related processes: knowledge-based interpretation and decision-making

Research focusing on reasoning-related PV processes has similarly found substantial qualitative differences between experienced and novice teachers. Novices’ reasoning is typically vague and often reflects judgmental evaluations or oversimplified assumptions about teaching and learning (Böheim et al., 2026; Farrell et al., 2022; Wolff et al., 2021). They struggle to provide analytical, knowledge-based explanations and predictions that are closely connected to principles of high quality teaching (Farrell et al., 2022; Sherin and van Es, 2009). Examining the think-aloud protocols of 67 teachers, Wolff et al. (2016) found that experts used complex language and deeper interpretations, suggesting they have a richer mental model of classroom dynamics, reflecting on both their advanced organizing and integrating capacities to make sense of the observed events. In contrast, novices often rely on less developed cognitive schemas that help categorize and interpret observed student behavior to make informed predictions about consequences for student learning. A study by Stahnke and Blömeke (2021) revealed that experienced teachers make more interpretative comments and suggest more alternative decisions for improved classroom management.

The role of schematic knowledge in expert reasoning is further illuminated by Wolff et al. (2021), who developed a theoretical model of classroom management scripts contrasting expert and novice teachers’ knowledge and awareness of classroom events. Their model proposes that expert teachers possess well-developed, hierarchically organized cognitive scripts for recurring classroom management situations, enabling rapid categorization of student behavior – directly reflecting the organizing process in the PV-CP model – and knowledge-based interpretation of its causes and consequences, reflecting the integrating process. Novice teachers, lacking such scripts, rely on more fragmented and superficial categorizations that limit their interpretive depth. Importantly, Wolff et al. (2021) also show that script-based knowledge shapes what teachers are aware of in the first place, providing an account of how reasoning-level structures feed back into noticing – consistent with the reciprocal architecture of the PV-CP model described in Section “1 Understanding teacher professional vision: a cognitive perspective.” It is therefore assumed that knowledge-based organization and integration processes construct well-developed mental models that promote elaborate reasoning about the observed teaching situation. In addition, studies indicate that, as a consequence of such well-developed mental models, experienced teachers demonstrate superior decision-making, for instance by providing more accurate diagnoses of student needs, which are essential for adaptive pedagogical actions (Chaudhuri et al., 2022; Seidel et al., 2021).

### 2.3 Summary: four characteristics of advanced professional vision

Taken together, empirical evidence suggests that experienced teachers exhibit more focused and efficient gaze patterns, better classroom monitoring, and higher judgment accuracy compared to novices. Summarizing insights from previous research, we identify four central characteristics of advanced PV. The first characteristic reflects the speed of information processing. Relying on well-developed scripts, experts rapidly notice subtle but relevant cues and immediately relate them to their instructional goals. Rather than being overwhelmed by the density of classroom events, they prioritize which information deserves further cognitive processing. Novice teachers typically process information in a bottom-up, saliency-driven way, attending to what is most visually striking and often overlooking subtle but instructionally relevant events in the classroom (Goldberg et al., 2021; Grub et al., 2020; Kosel et al., 2024). Second, advanced PV is characterized by knowledge-based noticing and reasoning. Based on a top-down regulation, experienced teachers draw on extensive professional knowledge to guide attention and interpret events within meaningful pedagogical contexts. They connect observed behaviors with declarative (e.g., curriculum goals, typical learning difficulties) and procedural knowledge (e.g., strategies for scaffolding participation), which allows them to recognize patterns, make predictions, and adapt their instruction accordingly. A third characteristic lies in the holistic use of the visual field. Whereas novices often focus narrowly on salient events, experts employ both foveal and parafoveal vision to monitor the classroom as a whole. They do not see isolated behaviors in fragments but perceive patterns and clusters of activity, akin to recognizing a whole “image chunk” rather than a collection of parts (see Pattern Recognition-Theory; Chase and Simon, 1973). This holistic perception allows them to anticipate emerging dynamics, such as a group of students drifting off task, before problems become disruptive. Finally, experts’ expanded working memory capacity enables them to hold and process a larger number of cues simultaneously (see Chunking-Theory; Miller, 1956). Through the development of retrieval structures in long-term memory, they can encode and interpret classroom events more efficiently than novices whose working memory is easily overloaded (Sweller, 2003), resulting in interpretations that remain largely descriptive.

In sum, advanced PV processes enable teachers to process information quickly, monitor classrooms holistically, and organize and integrate professional knowledge into coherent mental models of teaching and learning. This combination results in accurate judgments and adaptive pedagogical decisions that enhance instructional effectiveness and student learning. It is important to note that the PV-CP model does not equate expertise with years of teaching experience. Rather, expertise is defined in terms of characteristic process profiles: the ability to select and interpret information efficiently, to monitor the classroom holistically, and to organize and integrate observed events into coherent, adaptive mental models. This process-oriented operationalization of expertise aligns with recent calls in the field to move beyond proxy measures such as years of experience and toward theoretically grounded criteria (König et al., 2022; Cortina et al., 2024).

## 3 Professional vision processes in aligned and non-aligned teaching situations

The PV-CP model does not conceptualize noticing- and reasoning-related PV processes as uniformly applied across all classroom situations. Rather, a core theoretical assumption of the model is that professional vision requires flexible adaptation of visual and cognitive processing depending on the degree of alignment between the situation-specific context and the teacher’s activated professional schemata. Teaching situations vary in the extent to which incoming information confirms routinized expectations (aligned situations) or contradicts and extends them (non-aligned situations). Consequently, expertise in professional vision is reflected not only in efficient and largely automated processing in aligned situations, but – crucially – in the ability to adapt noticing and reasoning processes when teachers encounter unfamiliar or ambiguous classroom events. This adaptive flexibility captures how expert teachers regulate the balance between automated and deliberate processing, reorganize and integrate information, and modify existing knowledge structures when facing novel classroom situations. In this sense, the aligned/non-aligned distinction is not merely a contextual variation to be tested empirically, but constitutes a conceptual lens for understanding how advanced professional vision operates under varying situational demands.

Aligned teaching situations are those in which teachers and students engage in predictable classroom scripts grounded in routinized professional knowledge that teachers have acquired over time as cognitive dispositions (Blömeke et al., 2015; Shavelson and Stern, 1981; Seidel et al., 2024). An example of such a routinized and aligned interaction script is that students typically raise their hands before speaking during whole-class discourse (Böheim et al., 2020a,b). Whole-class discourse is commonly structured in three phases, in which teachers initiate the interaction by posing a question, students raise their hands to respond, and teachers provide feedback–known as the IRF pattern (Alexander, 2018; Böheim et al., 2022). In routine situations, teachers can rely on established cognitive scripts that enable efficient noticing and reasoning. For example, when monitoring students’ hand-raising behavior after posing a question, teachers can draw on familiar patterns of attention and response (Böheim et al., 2020a; Kosel et al., 2023). When preexisting scripts can be applied in routinized ways–such as when students are attentive, raise their hands, and give expected responses–teachers can process information rapidly and effortlessly by activating well-established schemata. This automation supports fast and efficient decision-making and allows cognitive resources to be allocated to other instructional demands.

However, classroom interactions rarely unfold entirely as scripted. Orchestrating classroom discourse is a complex task (Böheim et al., 2021, 2022; Osborne et al., 2013), and teachers often encounter unexpected or ambiguous student behavior that does not align with their activated scripts–such as disengagement, off-task behavior, or unanticipated questions. In such situations, teachers must engage in more effortful information gathering and allocate cognitive resources to analyze possible reasons for the non-expected student behavior. Teachers adapt their visual processing with a more focused attention on the problematic behavior, to identify contextual factors and make sense of the unexpected event (Wolff et al., 2016, 2017). In such non-aligned teaching situations, expertise differences might become particularly salient, as PV processes are no longer guided by automated scripts and schema activation needs to be adapted. Teachers must flexibly integrate new information, adapt and extend existing knowledge structures, and engage in more deliberate reasoning to interpret the situation accurately. Research on classroom management shows that experts can interpret problematic events with greater speed and diagnostic accuracy than novices (Wolff et al., 2021). Conversely, when teachers lack well-developed pedagogical knowledge, they may fail to notice problematic situations–such as signs of student boredom–and consequently miss opportunities for proactive intervention (Murtonen et al., 2022). Whether a teaching situation is experienced as aligned or non-aligned is itself shaped by contextual and environmental factors that lie beyond the intra-individual cognitive level modeled in the PV-CP framework. These factors influence the degree of fit between the situation-specific context and activated professional schemata, thereby influencing the extent to which processing can remain automated or switched to a more deliberate mode. The PV-CP model deliberately describes the cognitive processes underlying this adaptation, while leaving the contextual determinants of alignment unmodelled. Integrating this cognitive account with contextually embedded perspectives on teaching and professional vision (e.g., Gegenfurtner, 2020) “constitutes an important direction for future theoretical development.”

Taken together, the distinction between aligned and non-aligned teaching situations reflects a core theoretical claim of the PV-CP model: that professional vision is an adaptive cognitive system, not a fixed set of automated routines. Expert professional vision is characterized precisely by the capacity to shift fluidly between efficient, schema-driven processing and more deliberate, flexible reasoning depending on situational demands. This adaptive regulation – across both noticing and reasoning processes – is what the PV-CP model identifies as the hallmark of expertise, distinguishing it from a purely experience-based account. Novice teachers may demonstrate adequate processing in aligned situations but are expected to show substantially greater difficulties when familiar scripts cannot be applied, reflecting less flexible and less differentiated knowledge structures. The aligned/non-aligned framework thus provides a theoretically grounded and empirically tractable basis for capturing the full range of expert professional vision.

## 4 Training interventions for developing professional vision in relation to the PV-CP model

Next to describing expertise differences, the PV-CP model provides a principled framework for evaluating and designing professional vision training interventions: any intervention can be assessed in terms of which of the four processes it targets, through what cognitive mechanism, and with what expected effect on noticing or reasoning. A broad landscape of evidence-based professional vision interventions exists and has been comprehensively reviewed elsewhere, including prompt-based video analysis approaches, structured observation tasks, and cognitive-load-reducing instructional designs (Hamel et al., 2019; Tripp and Rich, 2012; König et al., 2022; Santagata et al., 2021). These approaches predominantly scaffold professional vision at the reasoning level – supporting the organizing and integrating processes through guided reflection, theoretical input, and structured knowledge activation. In this section, we focus specifically on Eye Movement Modeling Examples (EMMEs) and Verbal Expert Self-Explanations (VESEs) as illustrative examples of how the PV-CP model can guide intervention design. These formats are selected not because other approaches are less effective, but because they represent the only established intervention formats that operate directly at the noticing level of the model – intervening in visual information selection and breadth of the visual field through gaze visualization – while VESEs simultaneously scaffold the reasoning level. Together, EMMEs and VESEs constitute a dual-level intervention strategy that maps onto the complete PV-CP architecture, representing a novel direction in professional vision training that has not previously been grounded in an explicit cognitive process model. Research suggests that novice teachers can develop expert-like PV skills through structured training interventions designed to enhance both noticing and reasoning processes (König et al., 2022). The majority of these trainings reflect video-based interventions, where professional knowledge is activated through video analysis, theoretical input, instructional prompts, or structured reasoning tasks (e.g., Farrell et al., 2024; Martin et al., 2022, 2023; Prilop et al., 2021; Santagata et al., 2021; Sommerhoff et al., 2023). These interventions encourage novice teachers to draw connections between theoretical knowledge and observed teaching situations, thereby encapsulating their declarative knowledge and creating flexible cognitive schema grounded in practice-based experiences (Seidel et al., 2024). Meta-analytic evidence supports the positive effects of video-analysis training in improving preservice teachers’ ability to notice and interpret relevant classroom events (König et al., 2022; Santagata et al., 2021). For example, Farrell et al. (2024) found that a video-analysis training on tutoring instruction led preservice teachers to notice more relevant tutoring events and to provide more detailed, knowledge-based interpretations after the training phase.

In recent years, the growing use of eye-tracking technology in professional vision research has paved the way for new training formats. One approach involves using gaze replays as a reflective tool that visualizes teachers’ own eye movements during instruction (e.g., Coskun and Cagiltay, 2021; Kaminskienė et al., 2023). In the context of higher education, Kaminskienė et al. (2023) demonstrated that reviewing gaze replays and heatmaps enabled university lecturers to reflect on how they distributed their visual attention across students. Similarly, Coskun and Cagiltay (2021) found that gaze replays from mobile eye-tracking made university instructors aware of uneven gaze patterns. Both studies demonstrate the potential of gaze-based feedback as a valuable tool for fostering professional vision in teacher training.

Besides reviewing one’s own gaze, another approach uses instructional videos with an expert gaze overlay–known as eye movement modeling examples (EMMEs)–to help novices learn by observing how experts allocate their attention to relevant visual information (Emhardt et al., 2023; van Gog et al., 2025). Although visualizing teacher gaze is considered to hold great pedagogical value, a recent meta-analysis concludes that there are still limited empirical insights into how eye-tracking can be used as a training tool in teacher education (Keskin et al., 2024). EMME can be implemented through different forms of attentional guidance, such as superimposed gaze indicators or displays that perceptually reduce irrelevant information (e.g., spotlight- or blur-based designs; Jarodzka et al., 2012). These design variants illustrate that EMMEs primarily scaffold noticing-related processes by externally guiding information selection. Complementing this perceptual guidance, instructional approaches such as task-specific instructions or prompts direct attention more indirectly by activating cognitive schemata, thereby shaping knowledge-based visual processing without altering the visual stimulus (e.g., Gabel et al., 2023). Research on EMMEs has predominantly been conducted outside teacher education in domains such as sports, medicine, aviation, and STEM education (Emhardt et al., 2023). Findings from this research have supported the effectiveness of EMMEs in fostering attention guidance as well as task performance (van Gog et al., 2025; Xie et al., 2021). In the context of teacher professional vision training, EMMEs likely represent innovative training opportunities because they are directly relevant to the visual and cognitive processes underlying professional vision (see Figure 1). EMMEs employ signaling techniques, such as highlighting or blurring, and thus guide attention toward critical classroom events. This supports efficient noticing through strategic information selection, helping novices to reduce the amount of irrelevant information from sensory input. In addition, EMMEs promote efficient reasoning processes by minimizing extraneous cognitive load and preserving working memory resources, thereby creating capacity for elaborate schema construction and integration with prior knowledge (van Gog, 2021). Based on the dual-channel assumption, EMMEs are assumed to be especially effective when visual scaffolds are integrated with verbal scaffolds (e.g., gaze visualizations accompanied by explanations), leveraging the modality effect (see Mayer, 2014). It should be noted, however, that creating EMMEs requires access to (mobile) eye-tracking equipment, video recording infrastructure, and post-processing time to overlay gaze visualizations onto instructional video. When tailored individually – for example, providing teachers with gaze replay feedback from their own recorded lessons – the time investment is considerably higher. These practical demands may limit the scalability of EMME-based training, particularly in resource-constrained teacher education settings, and represent an important consideration for implementation research. Within the PV-CP framework, EMMEs primarily target noticing-related processes by scaffolding information selection and breadth of the visual field, while VESEs primarily target reasoning-related processes by supporting cognitive organizing and integrating; used together, they form a complementary intervention pair aligned with the model’s full process architecture. Research from example-based learning has drawn attention to the benefits of providing instructional (expert) explanations to promote learning and problem-solving (Renkl, 2002; Wittwer and Renkl, 2010). The benefits of verbal explanations are typically explained through principles of cognitive load theory (Sweller, 2003; Sweller et al., 1998), as they foster efficient cognitive organizing and integrating processes by reducing unnecessary cognitive load on working memory. Providing verbal expert self-explanations (VESEs), in which experienced teachers articulate their reasoning while analyzing classroom situations, is therefore assumed to complement the attention guidance provided by EMMEs by additionally scaffolding the reasoning-related processes of PV. Previous research shows that the combination of EMME and VESEs can effectively activate professional knowledge, scaffolding novices in structuring their observations and forming meaningful interpretations (Jarodzka et al., 2013; van Marlen et al., 2018). However, there are few studies that systematically investigate the effects of integrating experts’ verbalizations with EMMEs (exceptions: van Gog et al., 2009; van Marlen et al., 2022) and prior research sometimes shows inconclusive patterns (e.g., van Gog et al., 2009). Based on these theoretical reflections and the reviewed research evidence, combining EMMEs and VESEs appears to be a promising dual intervention strategy that unlocks the necessary visual and cognitive scaffolds required for effective professional vision training. However, to build a solid evidence base, it requires carefully designed experimental studies.

## 5 Scope and limitations of the PV-CP model

The PV-CP model offers a cognitive perspective on the central processes underlying teacher professional vision, its scope is deliberately circumscribed, and several limitations should be acknowledged. First, the model focuses on intra-individual cognitive processes and does not directly model the role of external contextual factors. Second, while the model distinguishes between novice and expert teachers as conceptual anchor points, it is important to acknowledge that teacher expertise lies on a continuum (König et al., 2022; Cortina et al., 2024). Within-group variability in professional vision is substantial, such that novice teachers may display advanced professional vision processes in particular situations. Fourth, the PV-CP model acknowledges that professional vision processes are not solely determined by cognitive knowledge structures, but are also shaped by teachers’ dispositional characteristics, including motivation, metacognition, and beliefs; however, these dispositions are not explicitly modeled as components of the framework. Given the limited empirical evidence on underlying mechanisms, these dispositional factors are treated as regulatory inputs that influence processing without being mechanistically specified. Finally, the empirical evidence for several process-level assumptions remains limited, underscoring the need for systematic experimental research directly testing the model’s predictions, particularly with respect to its four-process architecture and the aligned/non-aligned situation distinction.

## 6 Conclusion

In this conceptual paper, a current gap in research on professional vision is addressed: conceptualizing and modeling cognitive processes in teacher professional vision skills. Recent advancements in teacher research have contributed to robust knowledge on relevant PV components and their relevance for teacher competencies, such as the relationship between rather stable teacher dispositions and professional vision as a situation-specific skill, as well as the relevance of professional vision for decision-making and further teaching performance (Blömeke et al., 2015). A research gap, however, still exists regarding the clarification of underlying cognitive processes of noticing and reasoning of ongoing classroom events. Solid evidence has been gathered regarding expertise differences in professional vision and effective interventions for training professional vision skills. By modeling cognitive processes of professional vision in the PV-CP model, as introduced in this paper, we demonstrated that these previous research advancements can be solidly integrated. Thereto, we provide a conceptual alignment of teacher professional vision research with expertise research in educational cognitive psychology. By modeling professional vision as a system of four interrelated cognitive processes, the PV-CP model is closely related to the CTVE (Gegenfurtner et al., 2023; Gegenfurtner, 2024), with which it shares a multi-memory store architecture as established in cognitive psychology and the differentiation of perceptual and interpretive processing. The two frameworks address different theoretical questions: the CTVE specifies visual expertise across professional domains, while the PV-CP specifies how cognitive processes are organized within the situated structure of classroom teaching. The PV-CP thereby provides a framework that aligns cognitive expertise theory with the noticing–reasoning architecture of teacher professional vision research and offers a basis for empirical and instructional work on the adaptive nature of expert teaching. For further research, we propose testing the robustness of the PV-CP model by systematically varying conditions to address specific model components. For example, systematic variations in processed teaching situations such as aligned or non-aligned situations can help to test how the four PV processes operate under varying complexity conditions. In addition, we have proposed two examples of teacher training interventions such as EMMEs and VESEs, which can be explicitly related to the four modeled PV processes and might help to learn more about differential and targeted training effects on PV processes.
